# The Hypertrophic Cardiomyopathy Myosin Mutation R453C Alters ATP Binding and Hydrolysis of Human Cardiac β-Myosin*[Fn FN2]

**DOI:** 10.1074/jbc.M113.511204

**Published:** 2013-12-16

**Authors:** Marieke Bloemink, John Deacon, Stephen Langer, Carlos Vera, Ariana Combs, Leslie Leinwand, Michael A. Geeves

**Affiliations:** From the ‡ School of Biosciences, University of Kent, Canterbury CT2 7NJ, United Kingdom and; § Department of Molecular, Cellular, and Developmental Biology, University of Colorado, Boulder, Colorado 80309

**Keywords:** Actin, Cardiac Muscle, Cardiomyopathy, Fluorescence, Kinetics, Myosin, Homology Models, Protein Structure-Function, Sequence Alignment

## Abstract

The human hypertrophic cardiomyopathy mutation R453C results in one of the more severe forms of the myopathy. Arg-453 is found in a conserved surface loop of the upper 50-kDa domain of the myosin motor domain and lies between the nucleotide binding pocket and the actin binding site. It connects to the cardiomyopathy loop via a long α-helix, helix O, and to *Switch-2* via the fifth strand of the central β-sheet. The mutation is, therefore, in a position to perturb a wide range of myosin molecular activities. We report here the first detailed biochemical kinetic analysis of the motor domain of the human β-cardiac myosin carrying the R453C mutation. A recent report of the same mutation (Sommese, R. F., Sung, J., Nag, S., Sutton, S., Deacon, J. C., Choe, E., Leinwand, L. A., Ruppel, K., and Spudich, J. A. (2013) *Proc. Natl. Acad. Sci. U.S.A.* 110, 12607–12612) found reduced ATPase and *in vitro* motility but increased force production using an optical trap. Surprisingly, our results show that the mutation alters few biochemical kinetic parameters significantly. The exceptions are the rate constants for ATP binding to the motor domain (reduced by 35%) and the ATP hydrolysis step/recovery stroke (slowed 3-fold), which could be the rate-limiting step for the ATPase cycle. Effects of the mutation on the recovery stroke are consistent with a perturbation of Switch-2 closure, which is required for the recovery stroke and the subsequent ATP hydrolysis.

## Teaser

**Background:** R453C is a mutation in human cardiac myosin and is associated with a high incidence of sudden cardiac death.

**Results:** R453C alters few kinetic parameters, except for the conformational changes associated with ATP binding and hydrolysis.

**Conclusion:** The closure of switch-2 on ATP is disrupted by R453C.

**Significance:** This is the first detailed kinetic analysis of the motor domain of the human β-cardiac myosin carrying the R453C mutation.

## Introduction

Heart disease remains one of the major health problems of the western world, and inherited cardiomyopathies affect more than 1 in 500 individuals (1–3). These inherited myopathies are the leading cause of sudden death in young people (4, 5). Mutations that result in hypertrophic or dilated cardiomyopathies (6) have been reported in most of the sarcomeric proteins of the heart (6, 7), and ∼40% of these are found in the motor protein β-myosin (2). Despite the frequency of the mutations and the disease, understanding how the mutation results in the disease remains poorly defined.

There are many reasons for this, one being that the disease is often not manifest at birth but develops in the adolescent or adult, thus, separating the trigger of the disease development from the long term secondary and tertiary consequences of adaptation remains difficult. In addition, a major problem in defining the functional effect of the mutation in myosin is that patient material to test hypotheses is very limited. Mutations in myosin must alter its properties if it triggers disease development, yet *in vitro* studies of mutated human cardiac myosin from patients have been contradictory (8). This was due to the lack of a human recombinant expression system. Because of this, studies of myopathy mutations in myosin have used transgenic mouse cardiac models or non-cardiac myosins (9). The mouse as a model system is not ideal because the mouse heart predominantly expresses the faster α-myosin rather than the β-isoform that predominates in the human ventricle. In fact, it has been demonstrated that the hypertrophic cardiomyopathy (HCM)^5^ mutation, R403Q, produces the opposite functional effect if the mutation is placed into mouse α-myosin than was observed in human β-myosin (10). Other studies have expressed HCM-causing myosin mutations using a smooth or non-muscle myosin heavy chain background (11, 12). Together these studies have reported quite contradictory results and demonstrate the need to study the mutations in the relevant human β-myosin background.

The problem of expressing functional recombinant striated muscle myosin was solved by Winkelmann and co-workers (13, 14) who demonstrated that chicken embryonic skeletal myosin can be expressed and folded properly in embryonic chicken cardiac myocytes and in mouse skeletal muscle myotubes. We have exploited this method successfully to generate milligram quantities of human-muscle myosin motor-domains in mouse C_2_C_12_ cells (15, 16). We recently published the first detailed biochemical kinetic study of human cardiac α- and β-myosin motor domains (16). This study demonstrated that the human β myosin motor domain, as expected, had properties similar to β-myosin isolated from the rabbit, rat, and cow and that the α-isoform was quite distinct from the β-isoform. The α-isoform, despite 91% sequence identity to the β-isoform in the motor domain, had properties similar to those of the fast skeletal muscle isoforms.

More than 300 mutations in the motor domain of β-myosin have been reported to be linked to human cardiomyopathies (6). The mutations occur throughout the motor domain, the rod domain, and in the light chains. Understanding how such a wide variety of different mutations can result in similar disease phenotypes remains a problem of current interest. One hypothesis suggests that mutations that enhance myosin motor activity lead to HCM, whereas mutations that decrease motor activity result in dilated cardiomyopathy (17, 18). The ability to produce the human β-motor domain with disease-causing mutations now allows us to test such ideas. We present here the first detailed kinetic analysis of an HCM mutation (R453C) in the myosin heavy chain of human β-myosin. Several reports have shown that R453C is a malignant mutation associated with a high incidence of sudden cardiac death (19–21).

**FIGURE 1.**
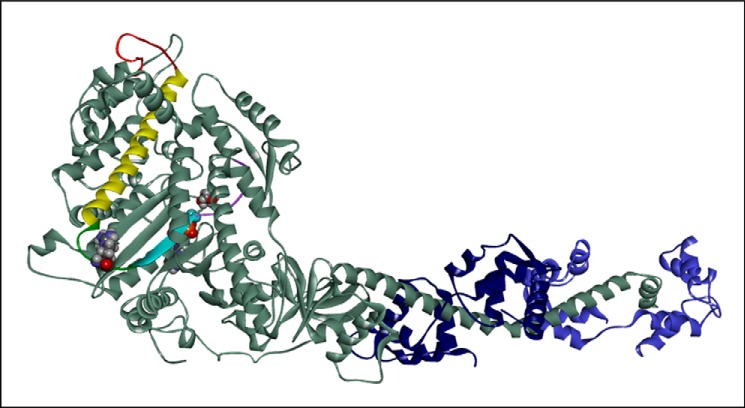
**Overview of cardiac myosin S1 head domain.** The S1 is shown with the essential and regulatory light chains (*ELC* and *RLC*) in *blue*. The location of HCM residue Arg-453 (*space-filling model*), the cardiomyopathy loop (*red*), and the long O-helix linking them (*yellow*) is indicated. Residue 453 is on the HO-linker (*green*) between the O-helix (*yellow*) and the central β5-sheet (*cyan*) + SW2 (*purple*). The location of the nucleotide is also shown as a *space-filling model*, close to SW2. The cardiac homology model was built using PDB code 1KK8 as a template (detached conformation, SW1 closed, SW2 open, melted SH1 helix).

Arg-453 is located in a surface linker of the upper 50-kDa domain of β-myosin known as the helix O (HO) linker. The position of the linker is shown in Fig. 1. This loop lies between two highly conserved structural elements. At the C-terminal side it is connected to switch-2 (SW2) of the nucleotide binding pocket via the fifth strand of the central β-sheet. At the N-terminal side it connects to the cardiomyopathy loop of the actin binding site via a long α-helix (helix O). The surface linker is, therefore, in a position to sense signals from both actin and nucleotide binding sites and affect the communication between these sites. The β-sheet strand (β5) and helix O are both highly conserved across the broad myosin family, whereas the HO-linker shows more variability between myosin classes. The HO-linker is identical among sarcomeric mammalian/vertebrate myosins II, but it differs in smooth and non-muscle myosin II (see supplemental Fig. S1). The HO-linker, therefore, appears to be specific for sarcomeric myosins, and the R453C mutation could lead to wide-ranging effects on the motor properties of the myosin heavy chain. In some myosin classes (*e.g.* myosin V and some myosin Is) there is a small insert between the β-sheet and the HO-linker. Of interest is the presence of a mutation (T380M) in this same linker in human mammalian myosin 1c associated with bilateral sensorineural hearing loss (22). We have shown previously that the T380M mutation is associated with wide-ranging changes in the binding and release of nucleotide by the acto·myo1C (23).

In closely related work, the effect of R453C mutation on the human β-myosin heavy chain was shown to reduce the actin-activated ATPase (by 20–30%) and inhibit *in vitro* motility (by 20–25%), whereas the single molecule step size was unchanged, and the intrinsic force was increased by 50% (24). We now report here a detailed evaluation of the nucleotide and actin binding properties of the R453C mutation in β-myosin S1 using stopped-flow. In contrast to the steady state and mechanical properties reported by Sommese *et al.* (24), under unloaded conditions we find small effects of the mutation on myosin heavy chain in our transient kinetic assays. Only the SW2 closure/ATP-hydrolysis step appears slower by at least 60% for the mutant compared with the ATP hydrolysis measured for WT. The implications of this change in the hydrolysis step for the myosin in the working heart are discussed along with structural models of the role of the Arg-453 residue in the WT myosin.

## EXPERIMENTAL PROCEDURES

### 

#### 

##### Proteins

Human β-S1^R453C^ was expressed and purified as described recently for human β-S1 wild-type (WT) (16). The R453C mutation was introduced into the shortβ-S1 sequence (Met-1–Arg-808) by QuikChange inverse PCR (Agilent Technologies, Inc.) using overlapping PCR primers containing a mismatch to produce the mutation. The presence of the mutation was verified by DNA sequencing. The mutation-bearing protein was co-expressed with the human ventricular essential light chain (MLC3) and when purified by this method was bound to MYL3 protein at a 1:1 ratio. This purified complex is referred to herein as β-S1^R453C^. Preparations of β-S1^R453C^protein assessed by SDS-PAGE were similar to those of β-sS1 from Deacon *et al.* (16). Rabbit skeletal muscle actin was prepared according to methods described previously (25) and labeled with pyrene at Cys-374 (26).

##### Transient Kinetics

All kinetic experiments were performed as described previously for human β-S1 (16) at 20 °C in 20 mm MOPS buffer, pH 7.0, with 100 mm KCl, 5 mm MgCl_2_, and 1 mm azide unless indicated otherwise. Measurements were performed with a High-Tech Scientific SF-61 DX2 stopped-flow system. Pyrene-actin fluorescence was excited at 365 nm, and emission was detected after passing through a KV389 nm cut-off filter (Schott, Mainz, Germany). Tryptophan fluorescence was excited at 295 nm and observed through a WG320 filter. The stated concentrations of reactants are those after mixing in the stopped-flow observation cell unless otherwise specified. Stopped-flow data were analyzed using the Kinetic Studio software provided by TgK Scientific as well as with Origin (Microcal).

**SCHEME 1.**
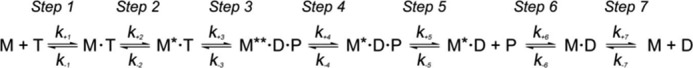
**The interaction of S1 with ATP and ADP.** S1, ATP, and ADP are represented as *M*, *T*, and *D*, respectively. * indicates the different levels of tryptophan fluorescence and represents different conformational states of the myosin (27).

**SCHEME 2.**
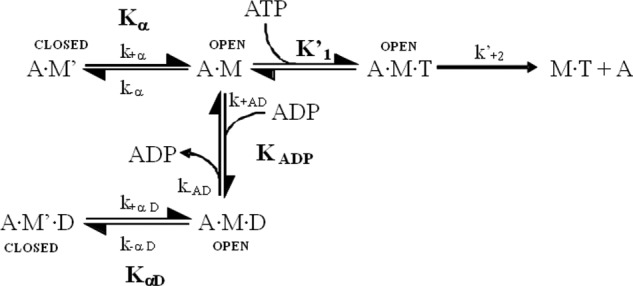
**The interaction of S1 with actin, ATP, and ADP.** *T* and *D* represent ATP and ADP, and actin·S1 exists in two conformations, A·M and A·M′, in equilibrium. A·M′ is unable to bind nucleotide and must isomerize to A·M before ATP can bind. A similar pair of conformations exist in the presence of bound ADP, where A·M′·D must isomerize to A·M·D before ADP can dissociate. Cross-bridge detachment from the rigor state (A·M) involves the complex binding ATP, governed by the association constant, *K′_1_*, followed by the rate-limiting isomerization, *k′*_+*2*_, after which actin-myosin affinity becomes weak, and the complex separates rapidly (28, 34).

Without actin present, the kinetics of S1 with ATP (T) or ADP (D) were interpreted based on the seven-step model described by Bagshaw *et al.* (27), where *k*_+_*_i_* and *k*_−_*_i_* are the forward and reverse rate constants, and *K_i_* (=*k*_+_*_i_*/*k*_−_*_i_*) represents the equilibrium constant of the *i*th step of the reaction (Scheme 1). In the presence of actin the kinetics of the interaction of S1 with ATP or ADP were analyzed based on the model previously developed for cardiac and slow myosin (16, 28–30). In Scheme 2, actin·S1 exists in two conformations (A·M and A·M′) in equilibrium. A·M′ is unable to bind nucleotide and must isomerize to A·M before ATP can bind. A similar pair of conformations exist in the presence of bound ADP, where A·M′·D must isomerize to A·M·D before ADP can dissociate. Pyrene-actin fluorescence is quenched in the pyrene·actin·S1 complex. Therefore, an increase in fluorescence intensity can be observed as a result of pyrene-actin dissociation from S1. The dissociation reaction can be induced by the addition of ATP to the pyrene·actin·S1 complex and resulting fluorescence transients analyzed to define the kinetics of the reaction. In the absence of ADP, high ATP concentrations induce the dissociation of actin from S1 in two phases. The fast phase represents dissociation from A·M according to Equation 1,

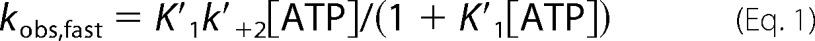
 The slow phase is limited by the isomerization from A·M′ to A·M with the maximum value of *k*_obs,slow_ = *k*_+α_. The ratio of the amplitudes of the fast and the slow phase of the ATP-induced dissociation defines the equilibrium constant between the two conformations in solution, and because *K*_α_ = *k*_+α_/*k*_−α_, *k*_−α_ can be determined using Equation 2,

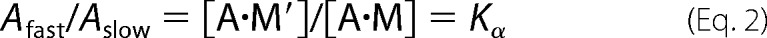
 For fast myosin S1, such as ^sk^S1 and ^pso^S1, *K*_α_ ≫ 1, and only a single phase is observed. In the presence of ADP, the overall dissociation constant of ADP for the actin·S1 complex (*K*_AD_) is defined by Equation 3,

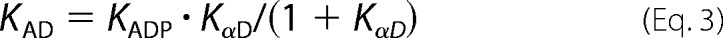
 where *K*_ADP_ is the dissociation constant for ADP from A·M and *K*_αD_ is the equilibrium constant between A·M′·D and A·M·D. If the binding of ADP is a rapid equilibrium event, controlled by *K*_ADP_, ADP and ATP compete effectively for binding to A·M, and the observed rate constant *k*_obs_ is defined by Equation 4,

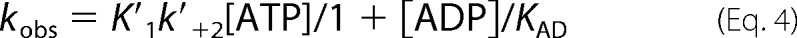
 The equation was used to analyze the data for ATP-induced dissociation of actin from the complex in the presence of ADP (see Fig. 4).

For the data shown in Fig. 5 the analysis of the titration of S1 binding to actin was performed as described by Kurzawa and Geeves (31). The amplitude of the observed transient is assumed to be proportional to the concentration of actin·S1 complex present before the addition of ATP. The data were fitted to the physically significant root of the following quadratic equation.

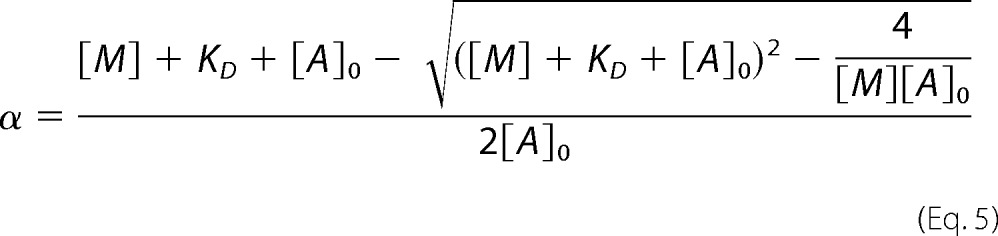
 For this equation, α is the fraction of actin with S1 bound, [*M*] is the total concentration of S1 added, [*A*]_0_ is the concentration of actin, and *K_D_* is the dissociation constant of S1 for actin in the presence of absence of ADP (*i.e. K*_A_ or *K*_DA_). In all cases the data in the figures refer to individual experimental measurements, whereas Table 1 gives the mean values of the fitted constant for two to three separate measurements using different protein preparations.

**TABLE 1:** **Summary of transient kinetic parameters measured for WT and β-S1^R453C^** Values are the mean ± S.D. based on a minimum of two or three different protein preparations. All measurements were performed at 20 °C in 100 mm KCl, 20 mm MOPS, 5 mm MgCl_2_, pH 7. All values are the average of at least two independent protein preparations and a minimum of three preparations for the numbers that show a significance difference to WT values. HC, human cardiac.

Measured parameter	HC β-S1*^a^*	HC β-S1^R453C^	Ratio β-S1^R453C/WT^
**ATP binding to acto-S1**			
*K*′_1_k*′*_+2_ (μm^−1^s^−1^)	1.6 ± 0.3	1.4 ± 0.2	0.85
*k*′_+2_(s^−1^)	1081 ± 50	1248 ± 136	1.15
1/*K*′_1_( = *k*′_+2_/*K*′_1_k′_+2_, μm)	675	891	1.32
*k*_+α1_ (s^−1^)	49 ± 12	69 ± 19	1.40
*K*_α_	12 ± 2	11	0.91

**ADP binding to acto-S1**			
*K*_AD_ (μm)	10 ± 3	18 ± 6	1.8
*k*_−AD_ (s^−1^)	64 ± 3	63 ± 2	0.98
*k*_+αD_ (s^−1^)	1.8 ± 0.1	2 ± 2	1.11
*K*_αD_	3.7 ± 0.4	8 ± 2	2.16

**Actin affinity**			
*K*_A_ (nm)	17 ± 7	24 ± 19	1.41
*K*_DA_ (nm)	229 ± 138	470 ± 4	2.05
*K*_DA_/*K*_A_	14	19	1.35

**ATP binding to S1:**			
*K*_1_*k*_+2_ (μm^−1^s^−1^)	1.5 ± 0.1	1.1 ± 0.2*^b^*	0.73
*k*_+2_ (s^−1^)	160 ± 23	102 ± 14*^b^*	0.63
1/*K*_1_ = *k*_+2_/*K*_1_*k*_+2_ (μm)	107	93	
*k*_slow_ = *k*_+3_ + *k*_−3_ (s^−1^)?	14	0.6–3.8*^b^*	0.14

**ADP binding to S1**			
*K_D_* (μm)	1.0 ± 0.1	0.7 ± 0.2	0.7
k_−_*_D_* (s^−1^)	0.56 ± 0.08	0.59	1.05
*K*_AD_/*K_D_*	10	26	

*^a^* Data are from Deacon *et al.* (16).

*^b^* Significant difference to WT *p* < 0.05.

##### Sequence Alignments and Homology Modeling

Three-dimensional homology models were generated for human β-S1 wild type and S1^R453C^ using the SWISS-MODEL automatic comparative protein modeling server (32, 33) as described previously for human β-S1 (16). Each of the available scallop myosin crystal structures was used as a template. Scallop structures were chosen because scallop is the only sarcomeric myosin crystallized in various conformational states of myosin during the cross-bridge cycle: the rigor, detached, post-rigor, and the ADP-bound near-rigor state and the pre-power stroke state (see Table 2).

**TABLE 2:**
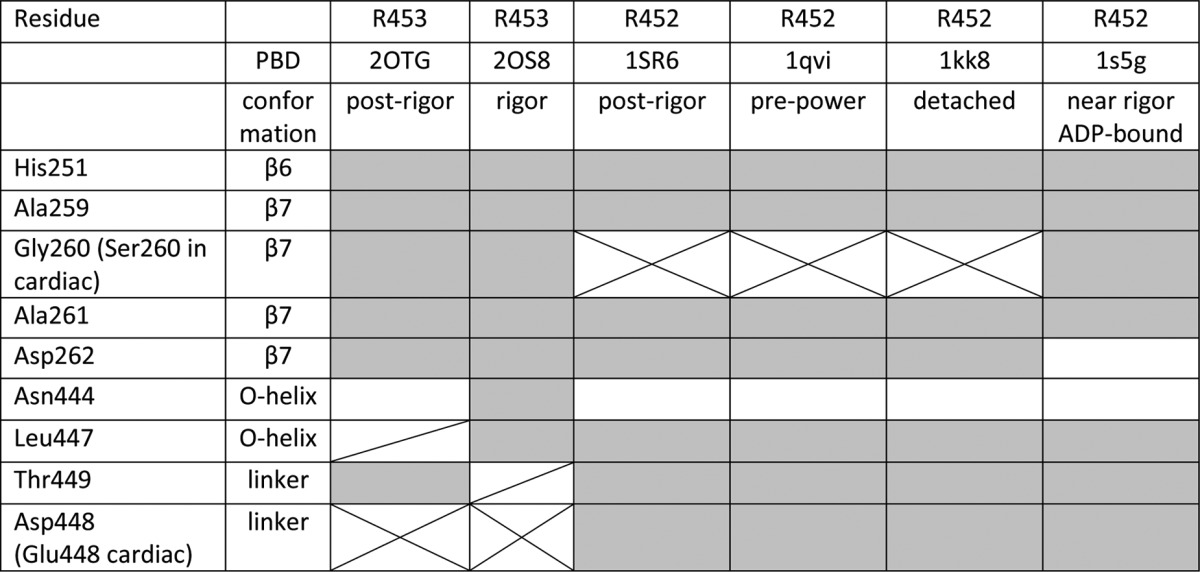
**Contacts between the side chain of Arg-453 and other residues close to the HO-linker for the various scallop structures and in homology models of human β-cardiac myosin** Grey, present in both the scallop structure and matching cardiac homology model; Cross, present only in the cardiac homology model; Diagonal, present only in the scallop structure; White, absent in both the scallop and cardiac homology model.

## RESULTS

### 

#### 

##### ATP Binding and Hydrolysis Is Slower for β-S1^R453C^

**FIGURE 2.**
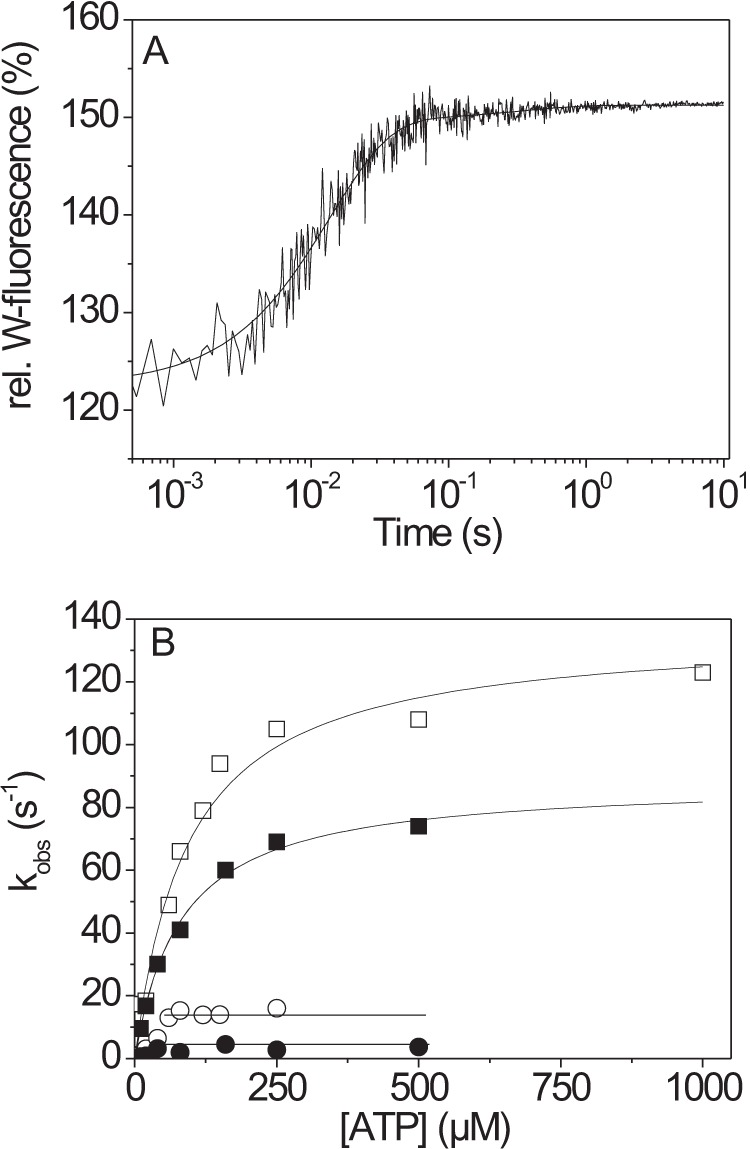
**ATP-binding to β-S1^R453C^.** *A*, 0.2 μm β-S1^R453C^ was rapidly mixed with 250 μm ATP using stopped-flow. The resulting change in tryptophan fluorescence could be best fitted using a double exponential from which the resulting *k*_obs_ was determined with *k*_obs_ = 69 s^−1^ for the fast phase (amplitude= 17%) and 1.3 s^−1^ for the slow phase (amplitude= 1%). *rel.W*, relative Trp. *B*, dependence of the fast phase *k*_obs_ on ATP concentration for β-S1^R453C^ (■) and β-S1WT (□) and their respective slow phases (●, ○). At high ATP-concentrations, *k*_obs_ (=*k*_+2_) saturate at 88 ± 3 s^−1^ for β-S1^R453C^ and at 137 ± 7 s^−1^ for β-S1 WT. From the hyperbolic fit, *K*_1_*k*_+2_ can be determined: *K*_1_*k*_+2_ = 1.08 ± 0.07 μm^−1^s^−1^ for β-S1^R453C^ and 1.5 ± 0.07 μm^−1^s^−1^ for β-S1 WT. At high ATP the observed slow rates (*k*_+3_ + *k*_−3_) saturate at 14 s^−1^ for β-S1 WT (○) and at 3–4 s^−1^ for β-S1^R453C^ (●). Note that the amplitudes of the observed reaction were independent of ATP concentration (fast phase 17 ± 1.5%, slow phase 1.2 ± 0.3%).

ATP binding to the β-myosin motor domain can be followed using the change in intrinsic tryptophan fluorescence. The highly conserved tryptophan on the end of the relay helix-loop provided the primary signal, and this is coupled to the rotation of the converter domain lever arm as part of the recovery stroke. The fluorescence change observed upon mixing 0.2 μm S1 with 250 μm ATP is illustrated in Fig. 2*A*. The signal has 2 phases: a large fast phase with amplitude of 17% and *k*_obs_ = 69 s^−1^ and a small slow phase, amplitude= 1% *k*_obs_ = 1.3 s^−1^. The observed amplitudes were independent of ATP concentration, whereas dependence of the *k*_obs_ values on ATP concentration are shown in Fig. 2*B*. The fast phase showed an approximate hyperbolic dependence on ATP concentration with *k*_max_ of 88 s^−1^, the ATP concentration required for *k*_max_/2 = *K*_0.5_ = 93 μm and initial slope of 1.08 m^−1^s^−1^. By analogy to our measurements of β-S1, these were assigned as: *k*_max_ = *k*_+2_, the initial slope = *K*_1_*k*_+2_, and *K*_0.5_ = 1/K_1_ (Scheme 1). The slow phase is difficult to measure at low ATP concentrations but above 50 μm is independent of ATP concentration. Again, by analogy with the wild-type data, this was assigned to *k*_+3_ + *k*_−3_, the rate constant of the ATP hydrolysis step, which is coupled with the recovery stroke. A summary of the data is shown in Table 1. For β-S1^R453C^ *k*_+2_ is reduced by ∼40% with respect to wild type. In these measurements the small variable amplitudes of the slow phase make it hard to measure with precision, but for β-S1^R453C^ it was consistent at, *k*_+3_ + *k*_−3_ ≤ 4 s^−1^.

##### Nucleotide Binding to Actin·β-S1^R453C^ Motor Domain Is Unchanged

**FIGURE 3.**
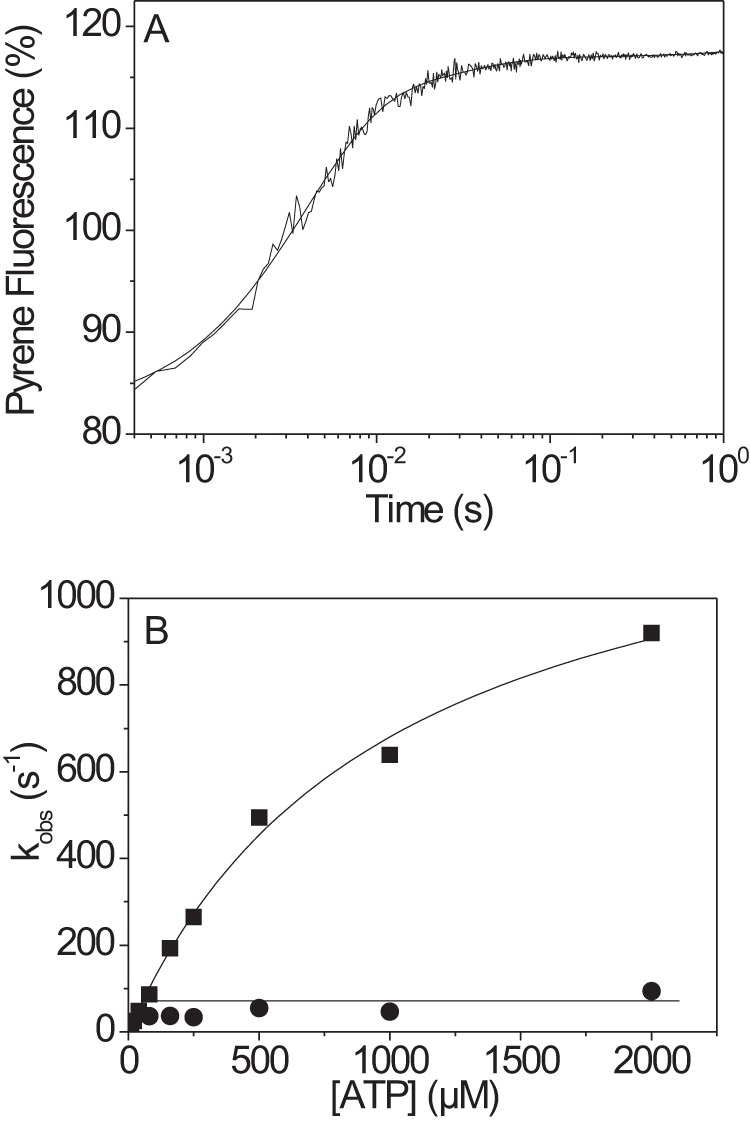
**ATP-induced dissociation of actin·β-S1^R453C^.** *A*, pyrene fluorescence trace observed upon rapidly mixing 0.05 μm pyrene-labeled actin·S1 with 250 μm ATP at 20 °C. The best fit was a double exponential with a fast phase (*k*_obs_ = 265 s^−1^, amplitude= 29%) and a slow phase (*k*_obs_ = 34 s^−1^, amplitude= 4.7%). *B*, dependence of *k*_obs_ on ATP concentration at 20 °C. At high ATP concentrations, *k*_obs_ saturates at 1357 ± 78 s^−1^ for the fast phase (■) and at 85 ± 20 s^−1^ for the slow phase (●).

ATP binding to actin·S1 results in rapid dissociation of actin from the complex as defined by Scheme 2. Fig. 3*A* shows the pyrene-actin fluorescence resulting from the rapid mixing of 50 nm actin·S1 with 250 μm ATP. A biphasic transient was observed with a large amplitude fast phase (29%, *k*_obs_ = 265 s^−1^) and a small (4.7%, *k*_obs_ = 34 s^−1^) slow phase. The *k*_obs_ of the fast phase was hyperbolically dependent upon ATP concentration (see Fig. 3*B*), and fitting Equation 1 to the data results in *k*′_+2_ of 1357 s^−1^ and 1/*K*_1_ = 891 μm. The slow phase, visible at [ATP] > 200 μm had a *k*_obs_ of 85 s^−1^ relatively independent of ATP concentration and was assigned to *k*_+α_ as for β-S1 WT (16). None of the differences between WT and R453C values for these constants was statistically significant (see Table 1).

**FIGURE 4.**
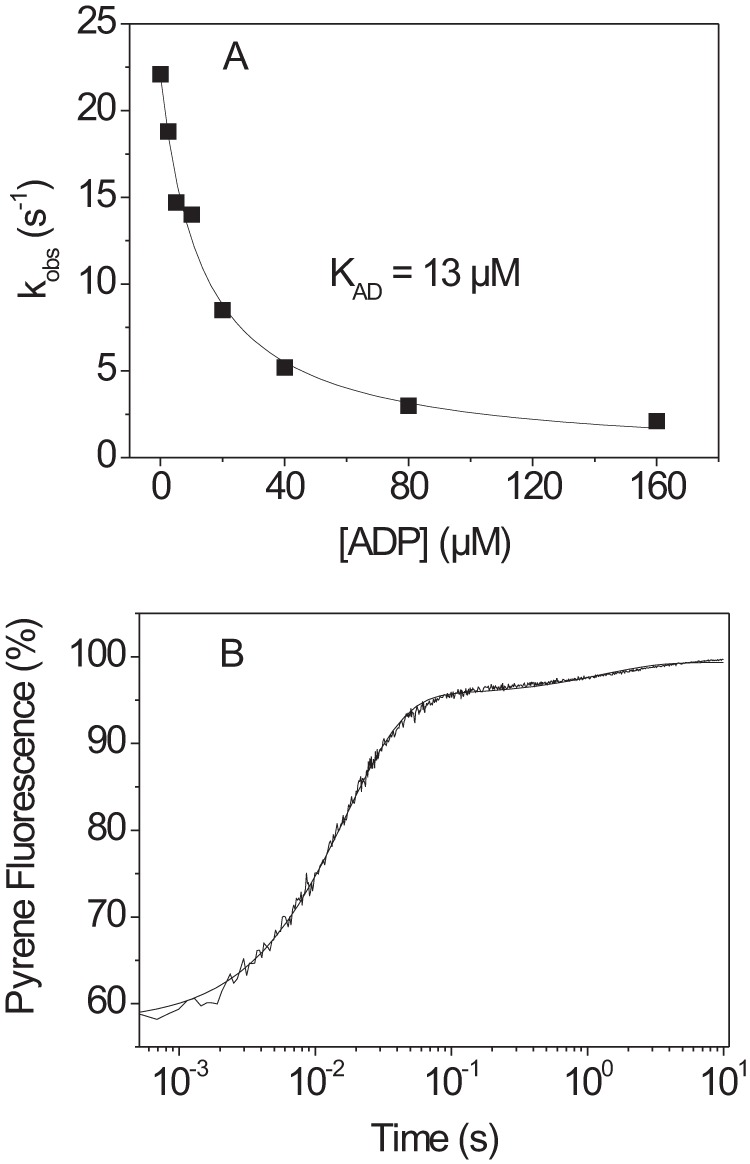
**ATP-induced dissociation of actin·β-S1^R453C^ in the presence of ADP.** *A*, 100 nm pyrene-labeled actin was incubated with an equimolar amount of β-S1^R453C^ before mixing with 20 μm ATP and variable ADP concentrations (20 °C). The data were fitted using a single exponential, resulting in an apparent affinity (*K*_AD_) for ADP (*K*_AD_ = 13 ± 1 μm). *B*, pyrene fluorescence trace measured for 100 nm actin·β-S1^R453C^ incubated with 100/50 μm ADP before mixing with 2 mm MgATP. The data are best fitted using a double exponential with *k*_fast_ = 60.4 s^−1^ (amplitude= 38%) and *k*_slow_ = 0.78 s^−1^ (amplitude= 4%).

At low ATP concentrations the dissociation reaction appears as a single exponential. Under these conditions the presence of ADP with the ATP acts as a competitive inhibitor as shown in Scheme 2. As ADP binding does not dissociate actin from the complex, the presence of ADP slows down the *k*_obs_ value as described by Equations 2 and 3. A plot of *k*_obs_ as a function of ADP concentration is shown in Fig. 4*A*, and fitting Equation 2 and 3 to the data defines the ADP affinity (*K*_AD_) as 13.1 μm, which is not statistically different from wild type.

When 100 nm actin·S1 was preincubated with 50 μm ADP to form the A·M·ADP ternary complex and then rapidly mixed with a large excess of ATP (1–4 mm) the *k*_obs_ for ATP-induced dissociation of actin from the complex is limited by the rate of ADP release from the complex. Fig. 4*B* shows the pyrene-actin fluorescence transient observed when using 2 mm MgATP. Two phases were observed with a *k*_obs_ of 84 (=*k*_−AD_) and 0.85 s^−1^(=*k*_−αD_) and amplitudes of 38 and 4%, respectively. The two phases were independent of the ATP concentration used in the range 1–4 mm ADP. The summary data are shown in Table 1, and the fast and slow phases were assigned to *k*_−AD_ and *k*_+αD_, respectively.

##### Affinity of Actin for β-S1^R453C^and β-S1^R453C^·ADP

**FIGURE 5.**
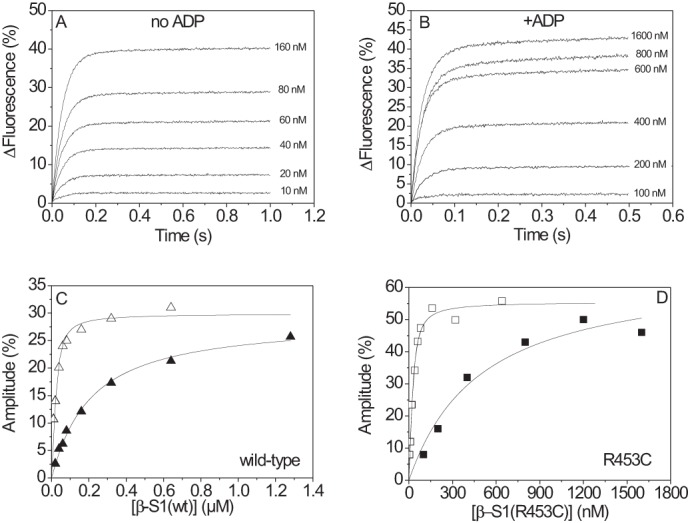
**Titration of actin with actin·β-S1^R453C^.** 30 nm phalloidin-stabilized actin was incubated with various amounts of β-S1^R453C^ before mixing with 20 μm ATP without ADP present (□) (*A*) or with 500 μm ATP in the presence of 100 μm ADP (■) (*B*). The fluorescence was fitted using a single exponential, and the amplitude increased with increasing S1 concentration. *C* and *D*, the best fit of the amplitude dependence on S1 concentration was determined using the quadratic equation describing the binding isotherm (see “Experimental Procedures”). This resulted in *K*_A_ = 8 nm (▵) and *K*_DA_ = 190 nm (▴) for β-S1 WT (*panel C*) and in K_A_ = 11 nm (□) and *K*_DA_ = 472 nm (■) for β-S1^R453C^ (*panel D*).

The affinity of actin for S1 was measured using the method of Kurzawa and Geeves (31). 30 nm phalloidin-stabilized pyrene-actin was incubated with various concentrations of S1 (0–160 nm) to form the actin·S1 complex. The complex was then rapidly mixed with 20 μm ATP, and the fluorescence transient was recorded. The amplitude of the transient was proportional to the concentration of complex present (see Fig. 5*A*). A plot of the amplitude against the total S1 concentration is shown in Fig. 5, *C* and D, for β-S1 WT and β-S1^R453C^, respectively. The data were fitted with the quadratic equation and defined the value of *K*_A_ = as 8 nm for WT and 11 nm for R453C. The measurement was repeated by incubating actin and S1 (0–1.6 μm) in the presence of 100 μm ADP to form the actin·S1·ADP complex. The transients observed on mixing rapidly with 500 μm ATP are shown for β·S1^R453C^ in Fig. 5*B* with the amplitudes plots shown in Fig. 5, *C* and *D*, for WT and R453C, respectively (*filled symbols* are in the presence of ADP). Analysis gave values of *K*_DA_ = 190 nm and 472 nm for WT and R453C, respectively. Table 1 shows the averaged results for a series of three measurements using different preparations of protein. Note that these measurements depend critically on the concentration of active protein in the sample and show more variation than the other measurements reported here. However, the ratio of *K*_DA_/*K*_A_ defines the degree to which ADP reduces the affinity of actin for S1, and the ratio corrects for any variation in active S1 concentration. Therefore, although the value of *K*_DA_ appears to be weaker for β-S1^R453C^, the *K*_DA_/*K*_A_ ratio showed no significant difference between WT and mutant.

## DISCUSSION

Table 1 summarizes the key differences in the data collected for the human β-S1^R453C^ compared with our earlier data for β-S1 WT. Few of the values show statistically significant differences between the two proteins. Only the values for ATP binding (*K*_1_*k*_+2_, *k*_+2_) and hydrolysis (*k*_+3_ + *k*_−3_) by S1 in the absence of actin can be shown to differ with any degree of confidence (*p* < 0.05). Normally for muscle type myosin IIs we expect to be able to define the values of each of the constants to an accuracy of 10–20%, but this does depend upon the stability of the protein and the size of the fluorescence changes measured in each step. In the case of β-S1^R453C^ and β-S1 WT, most values were defined to within 20%, with the errors being similar on both constructs. The exceptions are *K*_A_ and *K*_DA_. *K*_A_ and *K*_DA_ values are dependent upon knowing the concentration of active S1 construct, and this is not always defined precisely. The value of the ratio *K*_DA_/*K*_A_ when both constants are measured with the same protein preparation eliminates this source of error, and this value does not significantly change for the mutant *versus* the WT constructs.

The values of both the apparent second order rate constant of ATP binding (*K*_1_*k*_+2_) and the maximum value of *k*_obs_ (*k*_+2_) were reduced in the mutant protein by 30–40% (*p* < 0.05) with the value of 1/*K*_1_ not changing significantly. We have assigned the maximum observed rate (*k*_obs_) value of the fast phase fluorescence to *k*_+2_ based on analogy with the WT protein where two clear phases of the fluorescence changes were observed. For β-S1 WT, the fast phase reports ATP binding, and the slow phase reports the hydrolysis step (Scheme 1). For β-S1^R453C^ the amplitude of the second phase of the fluorescence change was small and difficult to measure with precision but where observed was much slower (<4 s^−1^) than for the WT (14 s^−1^). The reduction in the ATP hydrolysis step from 14 to 4 s^−1^ is a large change and may have a dramatic effect on the ability of the β-myosin to support cardiac contraction. We, therefore, evaluated carefully the assignment of the two components of the fluorescence change to ATP binding and hydrolysis.

Two observations suggest that *k*_max_ of the fast phase does reflect the ATP binding step. First, the amplitude of the observed fast phase showed little dependence upon the ATP concentration, and the amplitude was similar for β-S1 WT and β-S1^R453C^. As we know from classic work with myosin II, the amplitude of the fluorescent change for ATP binding decreases at high ATP concentrations if it monitors the hydrolysis step (34, 35). Second, ATP binding is irreversible in step 2 for all known myosin-II, and this predicts that the ATP concentration required for *k*_obs_ = *k*_max_/2 is different for the ATP binding step *versus* the hydrolysis step.

For two-step ATP binding the dependence of *k*_obs_ on ATP concentration is hyperbolic with Equation 6,

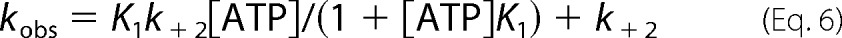
 For this system *k*_max_ = *k*_+2_, and the apparent second order binding constant is *K*_1_*k*_+2_ and *k*_obs_ = *k*_max_/2 when [ATP] = 1/*K*_1_.

For two-step ATP binding followed by ATP hydrolysis with the major fluorescence change on ATP hydrolysis, the second order binding constant is *K*_1_*k*_+2_ as before, with ATP binding irreversible (*k*_−2_ ∼ 0) and *k*_max_ = *k*_+3_ + *k*_−3_. Thus, *k*_obs_ = *k*_max_/2 will occur at the ATP concentration when *K*_1_*k*_+2_[ATP] = *k*_max_/2.

Note that the value of *K*_1_*k*_+2_ is the same in both models. Thus if *k*_max_ = *k*_+2_, we predict what the half maximum *k*_obs_ will be when [ATP] = 1/K_1_ = 93 μm as reported in Table 1. In contrast, if *k*_max_ = *k*_+3_ +*k*_−3_ = 102 s^−1^, then *k*_max_/2 = 51 s^−1^. [ATP] *K*_1_*k*_+2_ will then have a value of 51 s^−1^ when [ATP] = 46 μm, which is a factor of 2 lower than observed. Thus the fluorescence change is likely to be associated with the hydrolysis step and not the ATP binding step. This is consistent with the fluorescence change occurring on the same step for WT and mutant. This assignment as shown in Table 1 means that the value of 1/*K*_1_ does not change for the R453C mutant. The observation that the ATP-induced conformational change (linked to closure of switch 1 and switch 2) is reduced by ∼40% is compatible with the location of the mutation in the surface HO-linker connecting SW2 with the actin binding site. Furthermore, if the mutation results in disruption of the SW2 closure, this would have an effect on the hydrolysis step (which requires SW1 and SW2 to be closed for correct alignment of the active site residues; Ref. 36) and may explain why this step is more difficult to observe for R453C. Unfortunately, the quantities of S1 required for a quench-flow study preclude us analyzing the hydrolysis step further at this time, but measuring the hydrolysis step directly in both wild type and mutant remains essential to confirm our interpretation.

The role of the hydrolysis step, in addition to repriming the myosin for the next power stroke, is to define the minimum detachment lifetime for the motor. Once ATP binds and detaches a myosin cross-bridge from actin, the myosin cannot rebind until the ATP is hydrolyzed. Here our data suggest that this detached lifetime (1/(*k*_+3_ + *k*_−3_)) increases from 71 ms for β-S1 WT to ∼250 ms for β-S1^R453C^. This is a 3–4-fold increase in the detached part of the ATPase cycle and, if the attached part of the cycle were to remain unchanged, would predict a significantly reduced duty cycle (fraction of the ATPase cycle for which the motor stays attached) for the R453C motor. This might be expected to have a major effect on the operation of the motor in the heart. However, note that the duty ratio is already quite low for these sarcomeric myosins (∼0.1), and in normal operation, only a small fraction (5–20%) of the myosin heads are attached at any one time. An individual myosin will, therefore, only turnover a small number of times during each heartbeat. Thus in an individual heterozygous for the R453C myosin, the effects of such a change in the detached lifetime may only be felt when contracting under very high loads or at very high speeds.

In a recent study the same mutation (R453C in human β-S1) was studied using a set of complementary assays: steady state ATPase, *in vitro* motility, and laser trap (24). These studies reported that the *k*_cat_ for the ATPase was reduced by ∼30% at 23 °C (from 7.4 to 5 s^−1^). This is similar in value to the hydrolysis rate constant we report here for β-S1^R453C^ and suggests that the rate of the ATP hydrolysis step could be a major contributor to the *k*_cat_ value. This recent work also reported that motility was slowed by ∼25% from 800 to 610 (± 5%) nm/s at 23 °C. We have previously assigned the ADP release rate constant (*k*_−AD_) as the event that limits the maximum shortening velocity of a muscle or the motility assay (16, 24, 30), and our value of 64 s^−1^ for ADP release in the WT predicts a velocity of (*d*/*t*_on_) 660 nm/s, which is on the order of that reported. However, we see no significant change in the rate constant of ADP release from actin·S1 (*k*_−AD_) for β-S1^R453C^ compared with β-S1 WT. Both assays report an error of ∼5% in the values, but the absolute precision is likely to be less than this, and further measurements are required to resolve the differences here. Note that the motility assays (∼45 mm) and ATPase measurements (∼15 mm) use a lower ionic strength than the 100 mm used here, which is closer to physiological conditions. Sommese *et al.* (24) also reported that the force measured using a single molecule assay was enhanced by almost 50% compared with WT, and a similar result was noted in an ensemble force assay. All of our data are under unloaded conditions and, therefore, would not report such an effect. However, a load on the motor is expected to slow the cycle by inhibiting ADP release. The enhanced force coupled with an inhibition of ADP release could account for the slower motility if some small drag were present in the motility assay that we do not detect in our solution assay.

The Arg-453 residue is in a surface loop (HO-linker) as shown in Fig. 1. Examining the sequences of a range of myosins indicates that this HO-linker, and specifically Arg-453, is highly conserved among all sarcomeric myosin IIs including those of *Drosophila* and scallop (see supplemental Fig. S1). Neither the HO-linker nor Arg-453 is conserved between striated and smooth or non-muscle myosin IIs or indeed compared with other myosin families. This is illustrated for all human myosin IIs in a sequence alignment of the area around the HO-linker (supplemental Fig. S1) together with the sequence of human myosin 1b and 1c and other myosin family sequences. The sequence that precedes the highly conserved β5 strand is QPRQ(H/Y), and this becomes RQGAS in smooth and non-muscle myosin II. Some myosin 1s and some myosin Vs have inserts in this linker. Of significance is the human myosin 1c, which has a five-amino acid insertion in the HO-linker immediately before the β5 strand, and a mutation (T380M) in the insertion is associated with bilateral sensorineural deafness in humans (22, 23).

**FIGURE 6.**
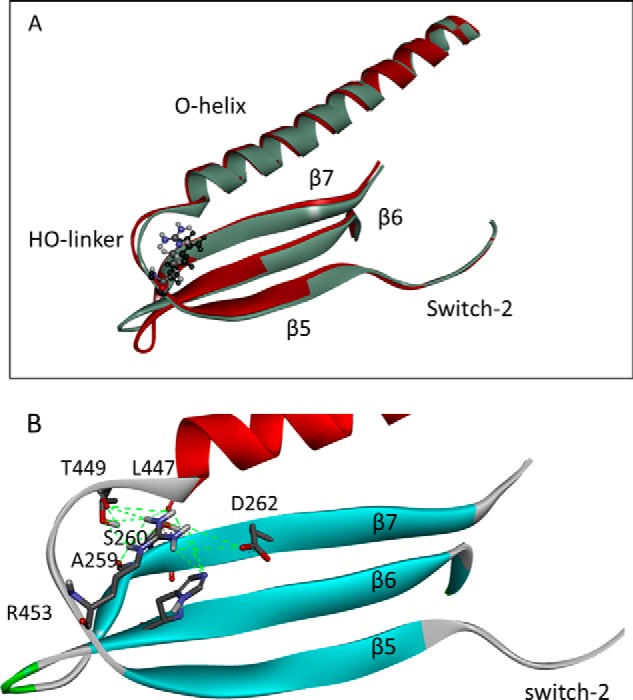
**Details of the HO-linker in scallop and cardiac myosin.** *A*, overlay of the crystal structures of cardiac myosin (PDB code 4DB1) and scallop (PDB code 1KK8) showing the O-helix, HO-linker, and β5, β6, and β7 of the central β-sheet. The cardiac structure is in *red*, the scallop structure is in *gray*, and the cardiomyopathy residue Arg-453 is shown as a *ball and stick model. B*, detail of the interactions of the Arg-453 side chain in a homology model of human cardiac myosin built using the scallop PDB 1QVI structure as template. A full list of the contacts between Arg-453 and the other elements is given in Table 2.

A crystal structure of the human cardiac β-myosin motor domain (PDB code 4DB1) allows us to examine the interactions of Arg-453 in the actin-detached state with AMP-PNP bound in the nucleotide pocket. These show the Arg-453 side chain is oriented toward the central β-sheet and has interactions with β-strand 6 (His-251), β7 (Ala-259, Ser-260, Asp-262), with residues located at the C terminus of the HO-helix (Asn-444, Ile-447) as well as the O-linker (Glu-448 and Thr-449) as shown in Fig. 6 (see Table 2 for a more detailed list of interactions). The substitution of the smaller uncharged Cys for Arg is predicted to lose most of these contacts (see supplemental Fig. 3), which presumably stabilize the relative orientation of the helix and the central β sheet.

This single conformation of the motor domain cannot tell us what happens as the motor goes through the major structural changes of the cross-bridge cycle. However, the high conservation of the Arg-453 in sarcomeric myosins II allows us to consider the interactions of Arg-453 in the other available crystal structures. Scallop myosin II is currently the best defined sarcomeric myosin. It has five defined structural states of the cross-bridge cycle: rigor (PDB code 2OS8), detached (PDB code 1KK8), post rigor (PDB codes 1SR6 and 2OTG), post-rigor ADP-bound (PDB code 1S5G), and pre-power-stroke (PDB code 1QVI). A sequence alignment of the three β-strands and the O-helix shows a high degree of conservation between scallop and human cardiac (supplemental Fig. 1). The scallop HO-linker retains the Arg, but the sequence adjacent to it deviates from the human cardiac sequence; QPRQY in human β-cardiac becomes AKRNY in scallop. The replacement of QP by AK could make the linker more flexible in the scallop myosin, so the models need to be examined carefully.

Fig. 6*A* shows an overlay of the human cardiac (PDB code 4DB1) and scallop (PDB code 1KK8) motor domain crystal structures in the region of the HO-linker. An overlay of the whole motor domain for the scallop and cardiac protein is shown in the supplemental Fig. S3 and indicates a good overall match between the two structures, particularly in the upper 50-kDa domain. The two structures in Fig. 6*A* match very closely; the only significant deviation is seen in the β-turn between β6 and β7 in which scallop has a Pro replacing the Ala in human cardiac myosin. The scallop myosin does, therefore, make a very good model of the human cardiac myosin structure in the area of the HO linker.

**FIGURE 7.**
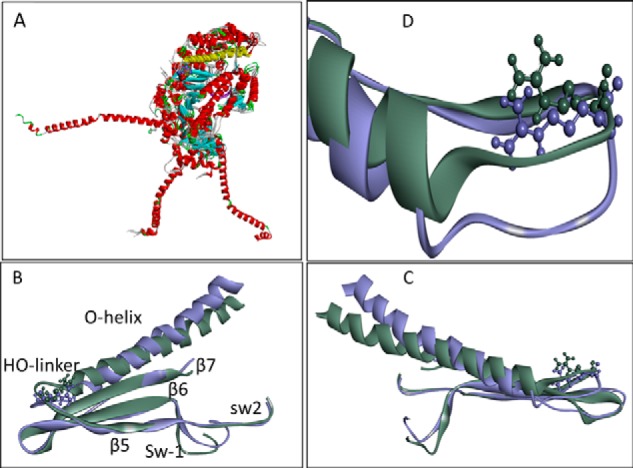
**Overlay of scallop crystal structures.** *A*, overlay of five Scallop crystal structures showing major reorientation of the converter and lever arm in the different myosin conformations. The changes in the 50-kDa domain are more subtle (*red ribbons* represent α-helices, *cyan arrows* are β-sheets. Note that the long O-helix (*yellow*) and central β-sheet (*cyan*) show only small movements. The Arg-453 area is shown in more detail in *B–D. B–D*, close-up showing the HO-linker movement between the rigor (PDB code 2OS8, *purple*) and post-rigor (PDB code 2OTG, *gray*) structures. The change from the rigor into the post-rigor conformation shows the biggest difference in the HO-linker conformation. The change involves closing of switch 1 and a twisting/bending of the O-helix. The orientation of *B* and *C* is 180° rotated. *D*, detail of the movement of the HO-linker. A list of the Arg-453 contacts in these structures is given in Table 2. Note that β6-β7 have been removed in *D* for clarity.

Overlaying all five of the scallop crystal structures shows that the upper 50-kDa domain is very similar in all conformations, the major differences being the converter and lever arm position as has been pointed out previously. Within the upper 50-kDa domain the orientation of the O-helix and the HO-linker with respect to the β-sheet remain to a first approximation unchanged (Fig. 7*A*) with small changes in the position of SW1 and SW2 and the cardiomyopathy loop in the actin-binding site. This section of the structure is shown in more detail for the two scallop structures (PDB codes 2OS8 and 2OTG) in Fig. 7, *B–D*. The β-sheet elements overlay very precisely, even though SW1 moves between the two conformations (Fig. 7, *A* and *B*). The distal (actin) end of the O-helix moves presumably as the CM loop binds/releases from to actin, resulting in a twisting of the helix and reorientation of the HO-linker. Examining the contacts between the Arg-453 side chain and the β-sheet, O-helix and HO-linker show remarkably little alteration in the number of contacts, although some distances do change. The Arg-453 contacts in each conformation of the scallop structures are listed in Table 2. This view of the HO linker is distinct from that reported for the *Dictyostelium* myosin 2, which has a different HO-linker sequence (see supplemental Fig. 1). In this structure a detailed molecular dynamics simulation shows extensive movement of the linker between different structural forms (see the supplementary movie in Preller and Holmes (38).

To test if these scallop structures were compatible with the cardiac myosin, we built five cardiac homology models based on the five scallop crystal structures. These all indicate a conformation of the HO-linker close to those in the equivalent scallop structure and similar contacts between Arg-453 and the β-sheet and O-helix (see Table 2). Of the nine contacts identified in the scallop structure, only four differ across the different structural states. The replacement of Gly-260 by serine in the cardiac muscle results in an extra contact between Ser-260 and the Arg-453 in some of the conformations (PDB codes 1SR6, 1QVI, and 1KK8). Similarly, the replacement of Asp by Glu-448 in cardiac allows an additional contact in post rigor and rigor conformations (PDB codes 2OTG and 2OS8). The contact with Leu-447 is lost in the cardiac equivalent of post rigor (PDB code 2OTG) and with Thr-449 in the rigor (PDB code 2OS8).

Thus overall the contacts that Arg-453 makes to the β-sheet, linker, and O-helix are retained in the cardiac β-myosin homology models. The conformations of the HO-linker seen in the scallop structure are possible for the cardiac myosin, and on current evidence these are the most likely structures to be adopted by the HO-linker. The scallop can be used as a guide to what the Arg-453 may do during the cross-bridge cycle.

In summary, Arg-453 has extensive contacts with the β-sheet, the O-helix, and the linker. Significantly, many of these contacts are maintained in all three structural states (rigor, post rigor, and pre-power stroke), which is consistent with the β-sheet (β5-β7), the HO-linker, and the O-helix moving together as a unit. The R453C mutation (or indeed the related R453S mutation (37)) would be predicted to lose most if not all of these H-bond contacts and, therefore, weaken the link between the loop, β sheet, and the helix. Indeed a homology model of the R453C cardiac myosin, based on the pre-power stroke conformation (PDB code 1QVI), showed only two of the eight contacts listed in Table 2 remain in place (Ser-260 on β7 and His-251 on β6; see supplemental Fig. 3). This loss of contacts may have a relatively minor effect in an unloaded cross-bridge cycle, but if the motor is bearing a load, this weakening of contacts could become much more significant. As stated above, the ADP release steps are known to be load-sensitive in myosins. The loss of the Arg-453 contacts may, therefore, affect the load dependence of the ADP release steps as indicated in the recent work of a single molecule study of the R453C mutation (24).

The two cross-bridge events that we find to be significantly affected by the mutation are the conformational change on ATP binding (step 2, *k*_+2,_ linked to SW1 closure) and SW2 closing/ATP hydrolysis step (step 3; *k*_+3_ + *k*_−3_). The R453C mutation is, therefore, in a position to influence or sense the movement of both switches and to influence how this is coupled to the upper 50-kDa domain through the O-helix. Arg-453 is just before β5, which joins the HO linker to SW-2. SW-2 closes onto the γ-P_i_ of ATP to trigger both the recovery stroke and the ATP hydrolysis step. Arg-453 makes significant contacts to β6, which, in the sequence, is preceded by SW1, which closes on the ATP to trigger opening of the 50-kDa cleft and actin dissociation. The R453C mutation could influence the closing of either switch (or the transmission of the switch closure to other parts of myosin) and, therefore, perturb ATP binding and/or the hydrolysis step. The loss of contacts between the HO-linker the central β-sheet and the O helix in the R453C cardiomyopathy is predicted to disrupt the coordinated movement of these central structural elements.

## Supplementary Material

Supplemental Data

## Footnotes

5 The abbreviations used are:

HCM: hypertrophic cardiomyopathy
S1: myosin subfragment 1
HO: helix O
SW2: switch-2
M: myosin motor domain
D: ADP
A: actin.
