# Supplementary material for: The Hypertrophic Cardiomyopathy Myosin Mutation R453C Alters ATP Binding and Hydrolysis of Human Cardiac β-Myosin
Source: J Biol Chem. 2013 Dec 16;289(8):5158–67. doi: 10.1074/jbc.M113.511204 (PMC3931073; doi:10.1074/jbc.M113.511204)
Supplement: Supplemental Data [file supp_289_8_5158__index.html]

The Hypertrophic Cardiomyopathy Myosin Mutation R453C alters ATP-binding and hydrolysis of human cardiac β-myosin — The Hypertrophic Cardiomyopathy Myosin Mutation R453C Alters ATP Binding and Hydrolysis of Human Cardiac β-Myosin — Effect of Cardiomyopathy Mutation R453C on Myosin Kinetics — Supplemental Data 

# The Hypertrophic Cardiomyopathy Myosin Mutation R453C Alters ATP Binding and Hydrolysis of Human Cardiac β-Myosin

## Supplemental Data

**Files in this Data Supplement:**

- Supplementary Figure 1 (.pdf, 750 KB) - Supplementary Figure 1
- Supplementary Figure 2 (.pdf, 202 KB) - Supplementary Figure 2
- Supplementary Figure 3 (.pdf, 237 KB) - Supplementary Figure 3
- Supplementary Figure 4 (.pdf, 314 KB) - Supplementary Figure 4
