# Supplementary material for: The Hypertrophic Cardiomyopathy Myosin Mutation R453C Alters ATP Binding and Hydrolysis of Human Cardiac β-Myosin
Source: J Biol Chem. 2013 Dec 16;289(8):5158–67. doi: 10.1074/jbc.M113.511204 (PMC3931073; doi:10.1074/jbc.M113.511204)
Supplement: Supplemental Data [file supp_M113.511204_jbc.M113.511204-1.pdf]

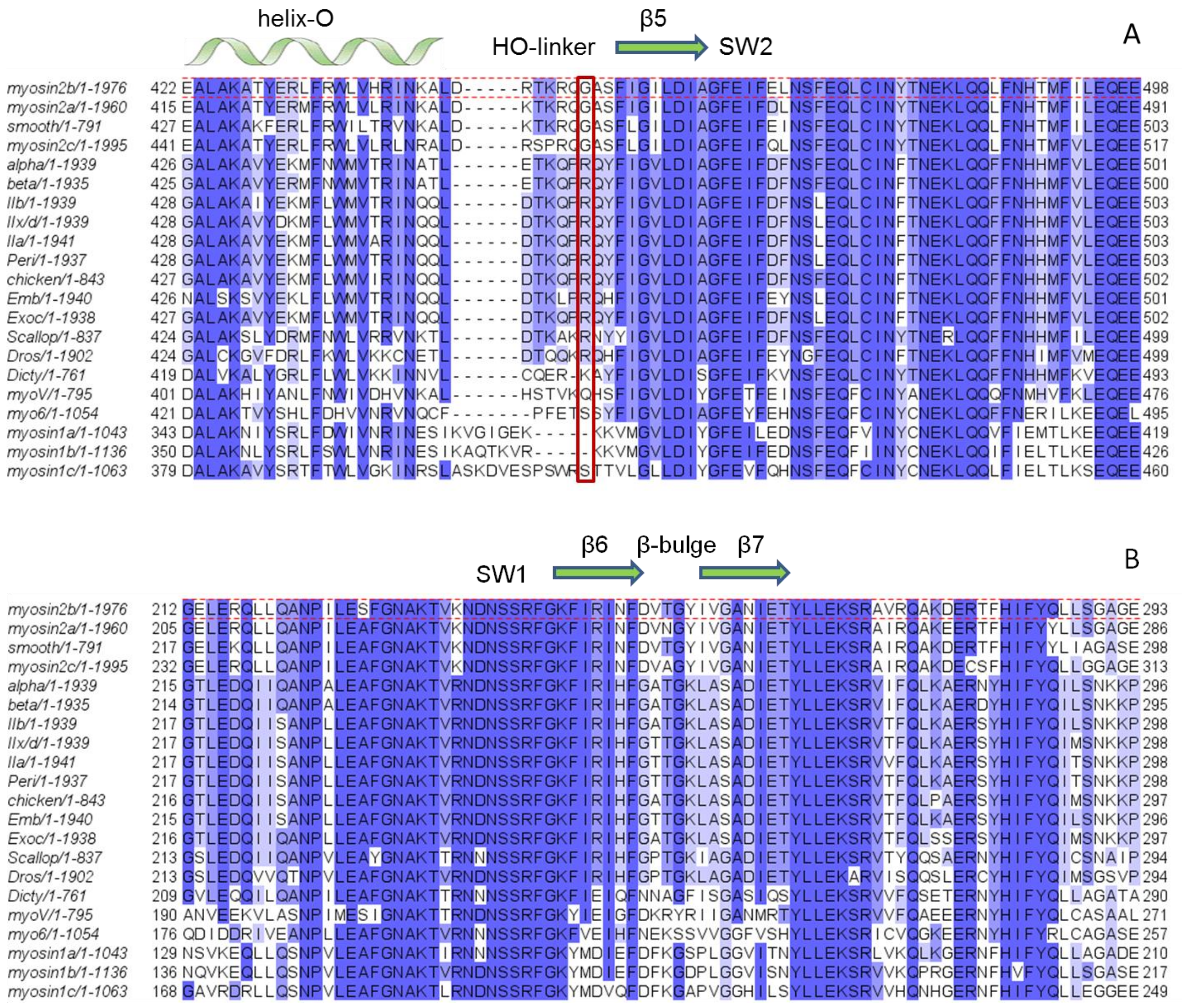

Figure S1: Alignment of myosin sequences near the R453 residue of human myosin II (A) or around SW1, β-6 and β-7 (B). All sequences are human unless otherwise labelled. The cardiomyopathy loop occurs just before the O-helix in the sequence (not shown). (A) R453 and the three elements O-helix, HO-linker and β5 are all highly conserved amongst the sarcomeric myosins II. (B) Alignment of the regions around SW1, β-6 and β-7 showing the high conservation of these areas across all myosins. The β bulge between the two β-strands is on the surface of the upper 50 KDa domain and lies between Loop 1 and the HO-linker and may contact one or both.
