# Supplementary material for: The Hypertrophic Cardiomyopathy Myosin Mutation R453C Alters ATP Binding and Hydrolysis of Human Cardiac β-Myosin
Source: J Biol Chem. 2013 Dec 16;289(8):5158–67. doi: 10.1074/jbc.M113.511204 (PMC3931073; doi:10.1074/jbc.M113.511204)
Supplement: Supplemental Data [file supp_M113.511204_jbc.M113.511204-2.pdf]

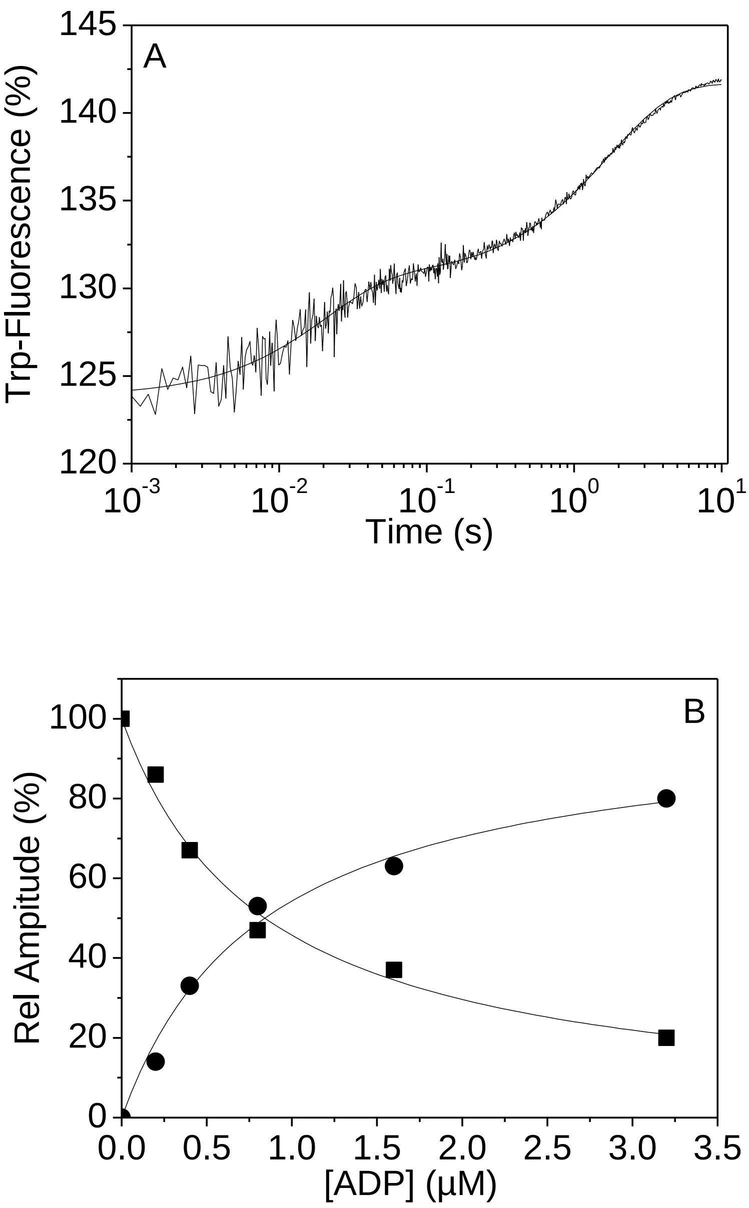

Figure S2: Displacement of ADP from S1^{R453C} by excess ATP. (A) The protein fluorescence changes observed after mixing 0.4 μM S1, pre-incubated with 0.8 μM ADP with 100 μM ATP (concentrations are before mixing). The fluorescence signal was fitted using a double exponential function with a fast phase (k_{obs} = 49.6 s^{-1} and amp = 4.7 %) and a slow phase (k_{obs} = 0.58 s^{-1} and amp = 7.8 %). (B) Dependence of the amplitudes of the fast (■) and slow phase (●) on [ADP], resulting in K_D = 0.84 μM.
