# Supplementary material for: The Hypertrophic Cardiomyopathy Myosin Mutation R453C Alters ATP Binding and Hydrolysis of Human Cardiac β-Myosin
Source: J Biol Chem. 2013 Dec 16;289(8):5158–67. doi: 10.1074/jbc.M113.511204 (PMC3931073; doi:10.1074/jbc.M113.511204)

Figure S3: Overlay of the human β-cardiac myosin crystal structure (4DB1) and scallop myosin-II crystal structure (1kk8) showing the whole myosin head. Cardiac myosin is in red, and scallop myosin-II is in grey. The O-helix is in yellow for cardiac myosin. The O-helix, HO-linker and central beta-sheet overlay very well for both structures.

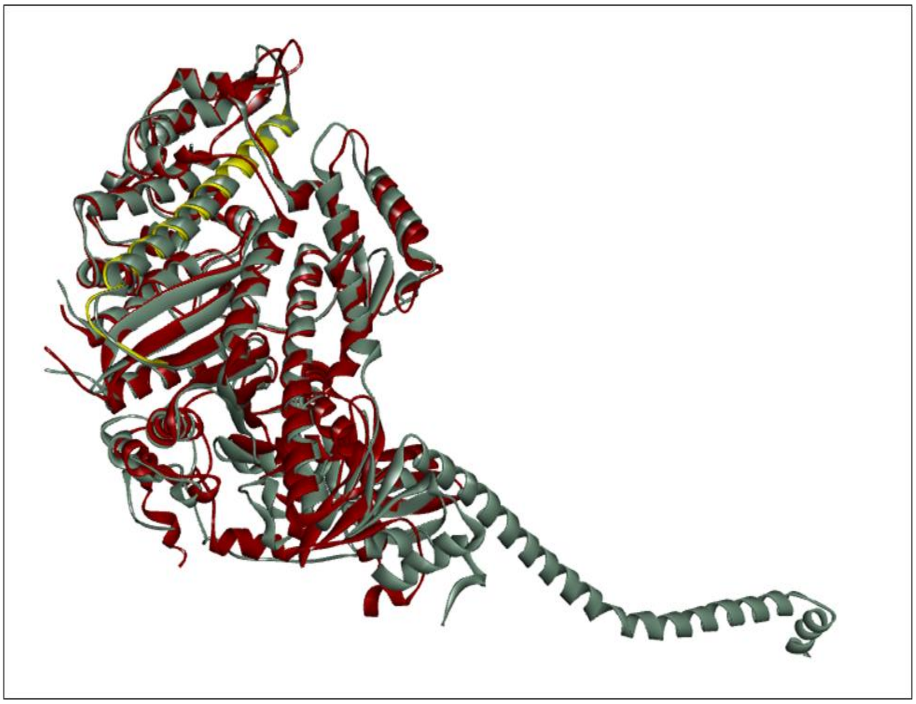

Supplement: Supplemental Data [file supp_M113.511204_jbc.M113.511204-3.pdf]
