# Supplementary material for: The Hypertrophic Cardiomyopathy Myosin Mutation R453C Alters ATP Binding and Hydrolysis of Human Cardiac β-Myosin
Source: J Biol Chem. 2013 Dec 16;289(8):5158–67. doi: 10.1074/jbc.M113.511204 (PMC3931073; doi:10.1074/jbc.M113.511204)

Figure S4: Homology model of the human β-cardiac myosin containing the mutation R453C. The Cys453 residue shows very few interactions with β6 (His251) and β7 (Ser260) and no interactions towards the O-helix are found (scallop 1QVI was used as template for this model)

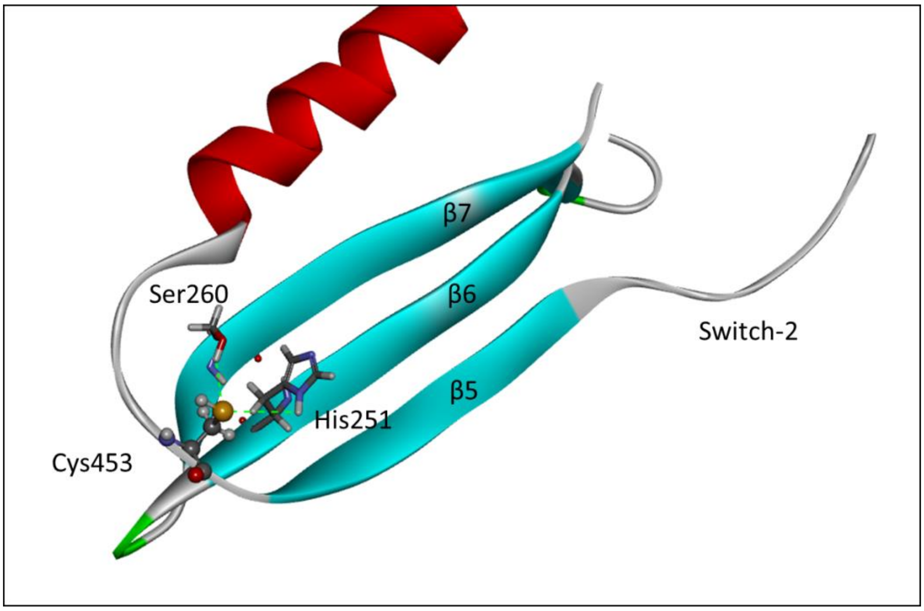

Supplement: Supplemental Data [file supp_M113.511204_jbc.M113.511204-4.pdf]
